# Clinical and radiographic evaluation of manus and pes lesions, including the carpus and tarsus, and associated risk factors in dromedary camels *(Camelus dromedarius)*

**DOI:** 10.3389/fvets.2026.1772182

**Published:** 2026-06-03

**Authors:** El-Sayed El-Shafaey, Madeh Sadan, Ahmed Ezzat Ahmed, Esam Mosbah, Fahd Al-Sobayil, Fahad A. Alshanbari, Walid Refaai

**Affiliations:** 1 Department of Surgery, Anaesthesiology and Radiology, Faculty of Veterinary Medicine, Mansoura University, Mansoura, Dakahlia, Egypt; 2 Department of Veterinary Surgery, Salam Veterinary Group, Buraydah, Qassim, Saudi Arabia; 3 Department of Clinical Sciences, College of Veterinary Medicine, Qassim University, Buraydah, Saudi Arabia; 4 Department of Biology, College of Science, King Khalid University, Abha, Saudi Arabia; 5 Prince Sultan Bin Abdelaziz for Environmental Research and Natural Resources Sustainability Center, King Khalid University, Abha, Saudi Arabia; 6 Department of Medical Biosciences, College of Veterinary Medicine, Qassim University, Buraydah, Saudi Arabia; 7 Department of Surgery, Anesthesiology and Radiology, Faculty of Veterinary Medicine, Zagazig University, Zagazig, El Sharkia, Egypt; 8 Department of Surgery, Anesthesiology and Radiology, University Veterinary Hospital, Qassim University, Buraidah, Saudi Arabia

**Keywords:** animals, diagnostic imaging, digit, lameness, pathology, radiology

## Abstract

**Aim:**

Injuries of the Manus and Pes, including the carpus and tarsus are a primary cause of lameness and subsequent economic loss in the camel industry. This retrospective study aimed to determine the prevalence of manus and pes lesions, including the carpus, and tarsus and evaluate the diagnostic utility of radiography in confirming clinical diagnoses of these conditions in dromedary camels. Furthermore, it investigated the risk factors associated with these injuries in the Qassim region.

**Methods:**

A total of 222 dromedary camels of various ages, sexes, and breeds were presented to the University Veterinary Hospital at Qassim University with manus and pes lesions, including the carpus and tarsus.

**Results:**

Clinical and radiographic assessments identified 325 distinct lesions. Hard-tissue lesions (62.5%)-predominantly fractures, joint dislocations, and bony exostoses, were significantly more prevalent than soft-tissue lesions (37.5%), such as fibromas, flexural deformities, wounds, and penetrating solar nails. These conditions were most frequently localized to the fetlock joint and the forelimbs. Key risk factors identified included juvenile age, female sex, body weight between 150 and 500 kg, and the Wadeh breed.

**Conclusion:**

Radiography is an essential diagnostic tool for identifying bone involvement, formulating targeted treatment plans, and establishing a reliable prognosis for the affections of the manus and pes, including the carpus and tarsus regions in camels when clinical examination alone is inconclusive. Identifying these risk factors will facilitate the development of preventative strategies to enhance camel welfare and herd productivity.

## Introduction

In dromedary camels, lameness is defined as an abnormal gait characterized by asymmetry or a non-weight-bearing posture on the affected limb, typically accompanied by pain. It represents a major welfare concern that adversely affects feeding behavior, social interactions, and overall productivity (1, 2). Lameness is a multifactorial condition resulting from the complex interaction of housing design, farm management, nutrition, and animal-related factors (3). Pathological conditions of the manus and pes, including the carpus and tarsus are among the most frequent causes of lameness in camels, leading to substantial economic losses for owners (4, 5).

Early and accurate diagnosis of manus and pes lesions, including the carpus and tarsus is critical for effective clinical intervention and the prevention of chronic lameness, which can severely compromise a camel's physical condition and performance (6, 7). Among available diagnostic tools, radiography remains a reliable, non-invasive, and widely accessible imaging modality in camel practice (8, 9). It provides essential, detailed information regarding the nature and severity of osseous lesions, enabling a definitive diagnosis in cases where clinical findings are inconclusive (10, 11).

While previous studies have documented various limb disorders in camels, including fractures, luxations, polydactyly, angular deformities, arthritis, osteitis, and osteomyelitis (11–13), the existing literature lacks comprehensive analyses specifically focused on the manus and pes lesions, including the carpus and tarsus and their associated structures. Furthermore, few studies have quantitatively evaluated the diagnostic value of radiography or clarified the specific risk factors predisposing camels to these lesions (2, 4). Therefore, the present study aimed to determine the prevalence of manus and pes lesions, including the carpus and tarsus in dromedary camels, evaluate the efficacy of radiography in refining clinical diagnoses, and identify the animal-related risk factors associated with these conditions. By examining a large dataset, this study provides systematic clinical and radiographic characterizations that strengthen diagnostic interpretation and offer new insights into the determinants of digit-associated lameness in camels.

## Materials and method

### Study design

A retrospective study was conducted on clinical cases presented to the Veterinary Hospital at Qassim University, Saudi Arabia, between January 2017 and August 2025. The study cohort consisted of 222 dromedary camels (*Camelus dromedarius*) of various breeds, both sexes, and ages, admitted specifically for the diagnosis and treatment of the manus and pes lesions, including the carpus and tarsus. For the purposes of this study, the manus and pes lesions, including the carpus and tarsus were evaluated from the proximal row of the carpal/tarsal bones to the distal phalanx. Inclusion criteria encompassed camels with lesions affecting the manus and pes, including the carpus and tarsus, including both soft-tissue and hard-tissue (osseous) disorders. Radiography was used to assess osseous involvement, while soft tissue diagnoses were based on clinical examination following thorough physical examination and typical gross presentation. Anatomical nomenclature followed the latest edition of the *Nomina Anatomica Veterinaria* (14–16). The study protocol was approved by the Animal Welfare and Ethics Committee of Qassim University.

### Clinical examination

A systematic clinical examination was performed on each camel by experienced camel clinicians. Initial observation was conducted at rest and during motion to subjectively evaluate gait and identify visual abnormalities of the manus and pes, including the carpus and tarsus. Lameness was graded on a scale of 0–3: Grade 0 (Functional soundness), Grade 1 (Mild), Grade 2 (Moderate), and Grade 3 (Severe) (17). This scoring system represents a non-validated clinical adaptation and a deviation from traditional bovine protocols necessitated by the species' unique movement patterns. The grading scale is based strictly on functional movement, regardless of the underlying pathology (Table 1). For detailed physical examination, camels were restrained in sternal recumbency and sedated with intravenous xylazine HCl (0.2 mg/kg; Seton 2%, Laboratorios Calier, S.A., Barcelona, Spain) as required by the animal's temperament. Once sedated, camels were positioned in lateral recumbency for thorough palpation and inspection of the affected limb, using the contralateral limb as a healthy control.

**Table 1:** Functional lameness grading and lesion distribution of the manus and pes, including the carpus and tarsus among the examined dromedary camel cohort.

Lameness grade	Frequency (*n*)	Percentage	Clinical gait characterization (functional assessment)	Clinical and radiographic findings (pathological assessment)
Grade 0 (functional soundness)	20	6.15%	Normal gait; rhythmic pacing; symmetrical weight bearing; no detectable gait deviation	Congenital structural anomalies (e.g., polydactyly, brachydactyly), mild footpad hyperkeratinization without pain
Grade 1 (mild)	62	19.08%	Subtle gait irregularity at a walk; identifiable shortening of stride or “stiff” movement apparent at a trot	Soft tissue lesions (fibromas, digital dermatitis), bursitis, superficial lacerations, chronic scarred wounds
Grade 2 (moderate)	65	20%	Obvious lameness at both walk and trot; consistent head bob or pelvic hitch; shortened weight-bearing phase	Claw/nail abscesses, chronic solar ulcers, non-septic arthritis, bony exostoses, flexural deformities, tendon lacerations
Grade 3 (severe)	178	54.77%	Marked reluctance to move; non-weight bearing at rest or during motion; extreme compensatory movements	Fractures, luxations, septic arthritis, gangrene, solar avulsions, deep foreign body penetrations, osteomyelitis
Total	**325**	**100**%	–	–

The classification above represents the most frequent clinical presentation observed in this cohort. Certain conditions, particularly abscesses, myiasis, and nail overgrowth, may span multiple grades depending on the depth of tissue involvement, secondary infections, or chronicity of the lesion.

A standardized questionnaire recorded data on potential risk factors: age, sex, body weight, and breed. Clinical parameters, including the affected limb (fore vs. hind), primary tissue involvement (soft vs. hard), presence of swelling, and number of distinct lesions, were documented. Furthermore, prognosis and treatment outcomes were recorded and systematically scored to create a clinical index for analysis (Table 2).

**Table 2:** Description and level for factors associated with surgical affections in a 222-camel cohort.

No.	Variable	Description and level
1	Age	Calves = 1; Juvenile = 2; Adult = 3
2	Gender	Female: 1; Male: 2
3	Body weight	< 200 kg = 1; 200 ≤ 500 = 2; > 500 = 3
4	Breed	Wadeh = 1; Mejhem = 2; Ashaal = 3; Asfar = 4
5	Acquisition	Congenital = 1; Acquired = 2
6	Affected limb	Forelimb = 1; Hindlimb = 2; Other region = 3
7	Presence of swelling	No = 1; Yes = 2
8	Target tissue	Soft tissue = 1; hard tissue = 2
9	Number of affections in each camel	One affection = 1; Two = 2; Three = 3; Four = 4; Five = 5; More than 5 = 6
10	Response to treatment	Responded =1; Failed = 2

### Radiographic examination

Radiography was employed for the definitive assessment of manus and pes lesions, including the carpus and tarsus. Standard projections, including dorsopalmar/dorsoplantar, lateromedial, and relevant oblique views of the affected regions, were obtained using a Min-X-ray HF 100/30 generator (Toshiba, Japan). Exposure settings were typically 70 kVp and 2.0 mAs with a 70-cm focal-film distance. Radiographs of the contralateral, clinically normal limb were routinely obtained to serve as a comparison control, following the method described by El-Shafaey et al. (18). An experienced clinician evaluated all radiographs to characterize the lesions before determining the final treatment plan.

### Statistical analysis

Data were compiled in Microsoft Excel 365 and analyzed using SPSS Statistics (Version 21, IBM, USA). Descriptive statistics determined the frequency and distribution of lesions. Differences in frequency were assessed using a Chi-squared test, and associations between lesions and clinical signs were evaluated using a Chi-squared test for linear trend.

Associations between manus and pes lesions, including the carpus and tarsus and hypothesized risk factors were analyzed via logistic regression. Initially, bivariate logistic regression was performed with the lesion presence as the dependent variable and risk factors as independent variables. Significant factors were then included in a multivariate, backward stepwise logistic regression model. Results are presented as regression coefficients, standard error, degrees of freedom, *p*-values, odds ratios (OR), and 95% confidence intervals (CI). A *p*-value < 0.05 was considered statistically significant.

## Results

### Clinical and radiographic findings

**Figure 1:**
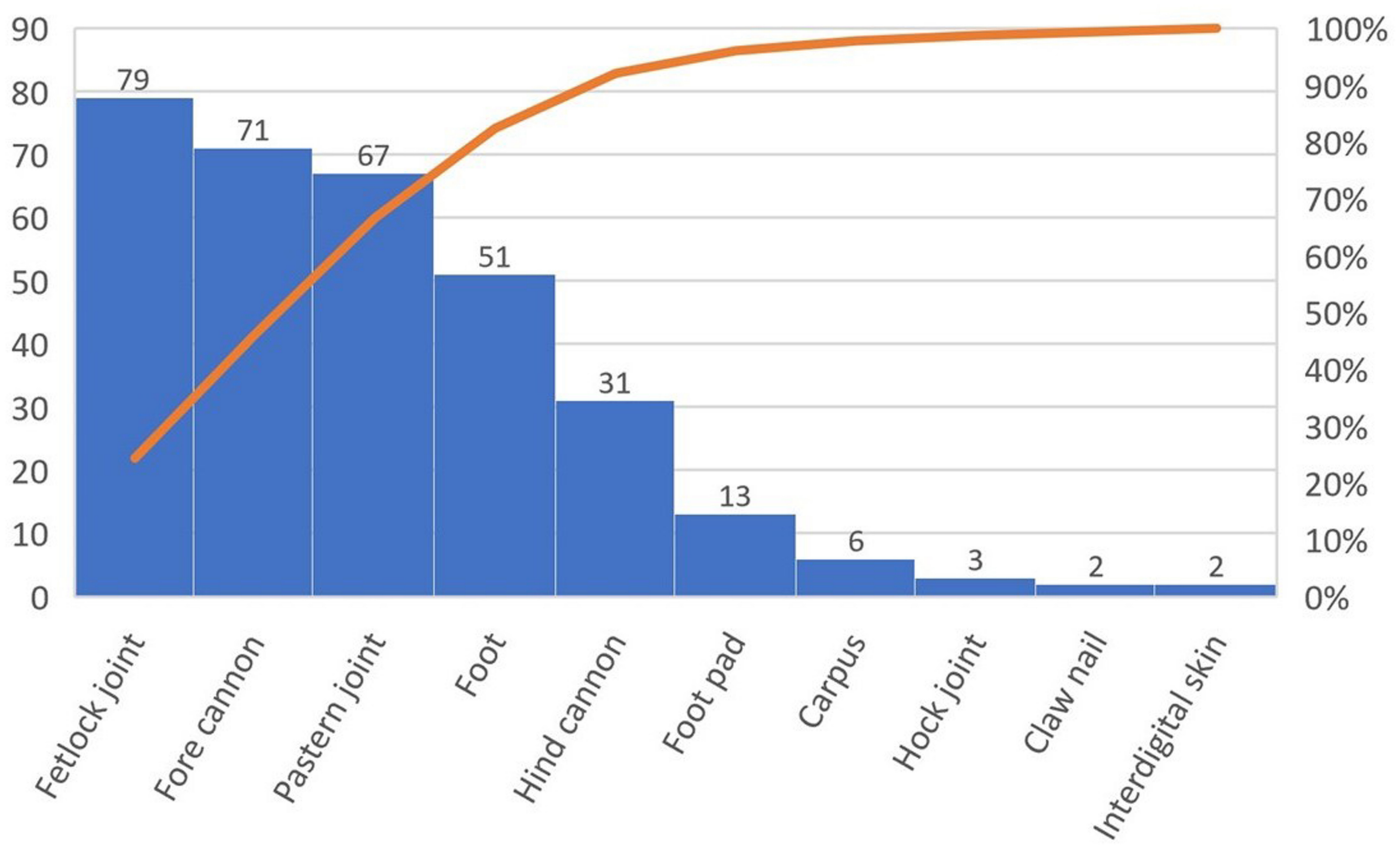
Prevalence and categorization of manus and pes lesions, including the carpus and tarsus. Bar chart illustrating the frequency distribution of 325 surgical conditions identified within a cohort of 222 dromedary camels in Saudi Arabia (2017–2025).

**Figure 2:**
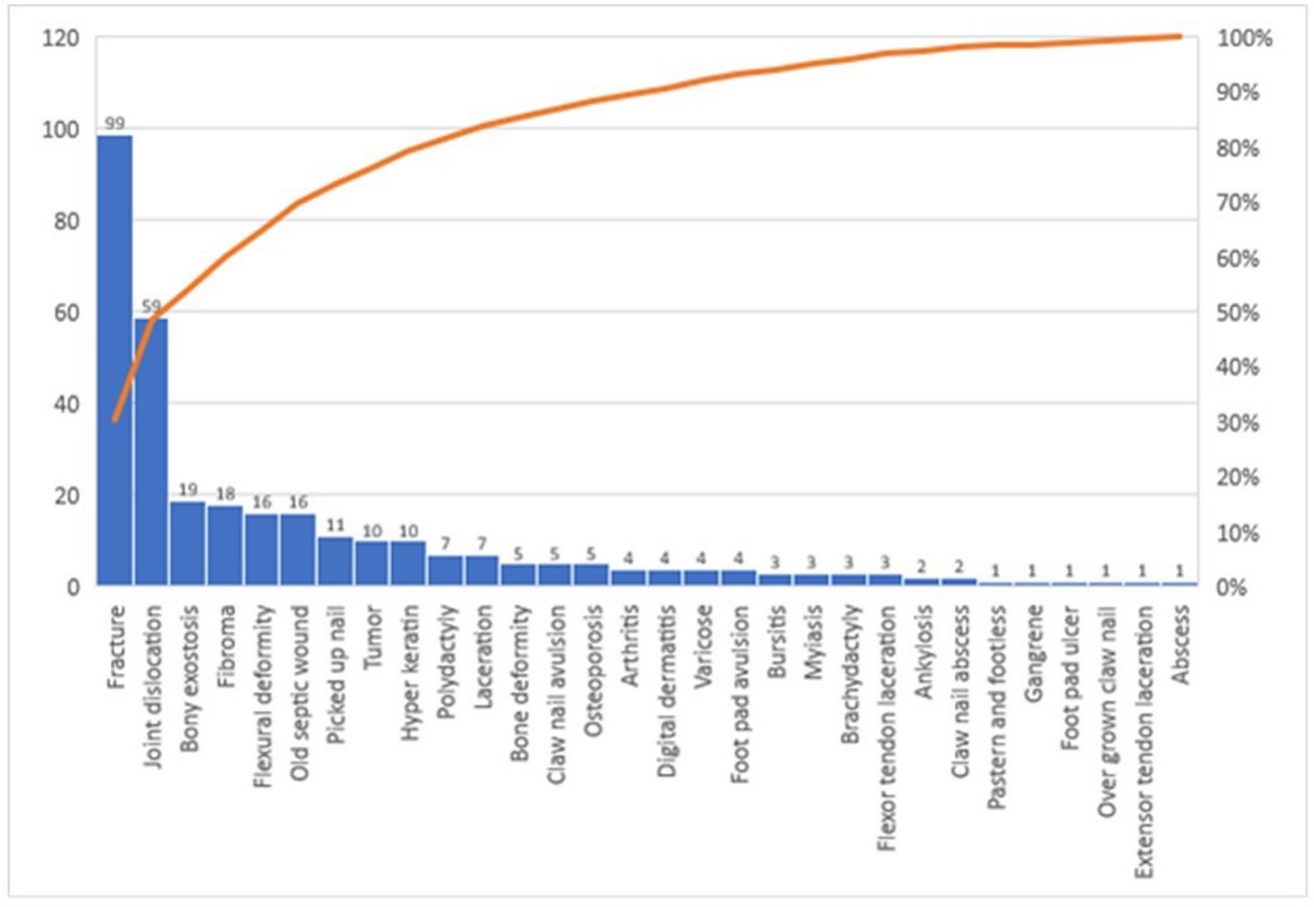
Anatomical distribution of lesions. Bar chart indicating the specific locations of 325 surgical affections in a 222-camel cohort examined in Saudi Arabia from 2017 to 2025.

**Figure 3:**
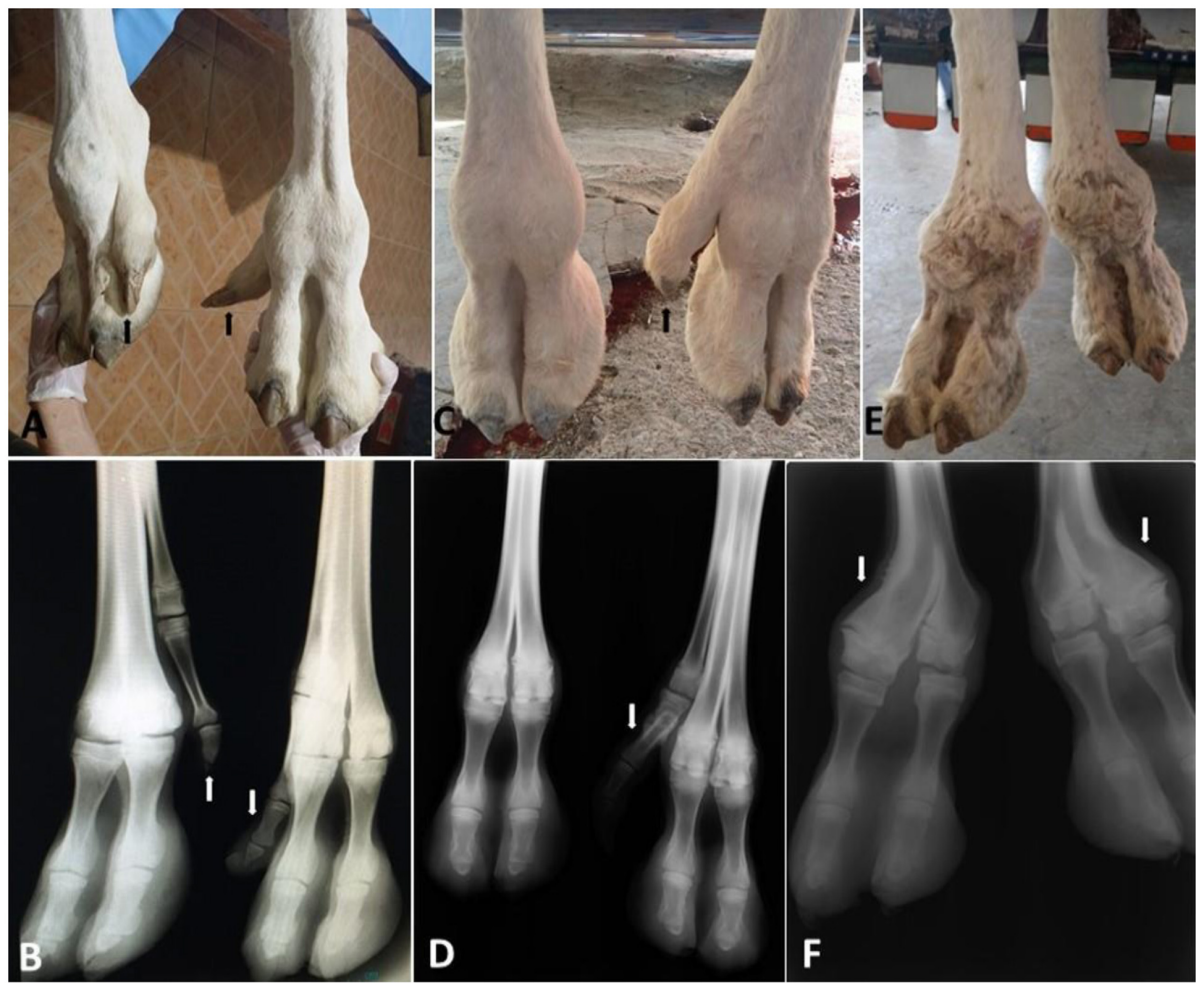
Congenital digital and bone deformities. **(A–D)** Polydactyly in Wadeh camel calves: **(A, B)** bilateral and **(C, D)** unilateral presentation of a complete supernumerary digit containing three phalanges (arrows). **(E)** Clinical view of an angular bone deformity at the fetlock joint in a 4-month-old calf. **(F)** Dorsopalmar radiograph of the same calf, revealing significant medial arching at the metaphyseal region of the metacarpus (arrows).

**Figure 4:**
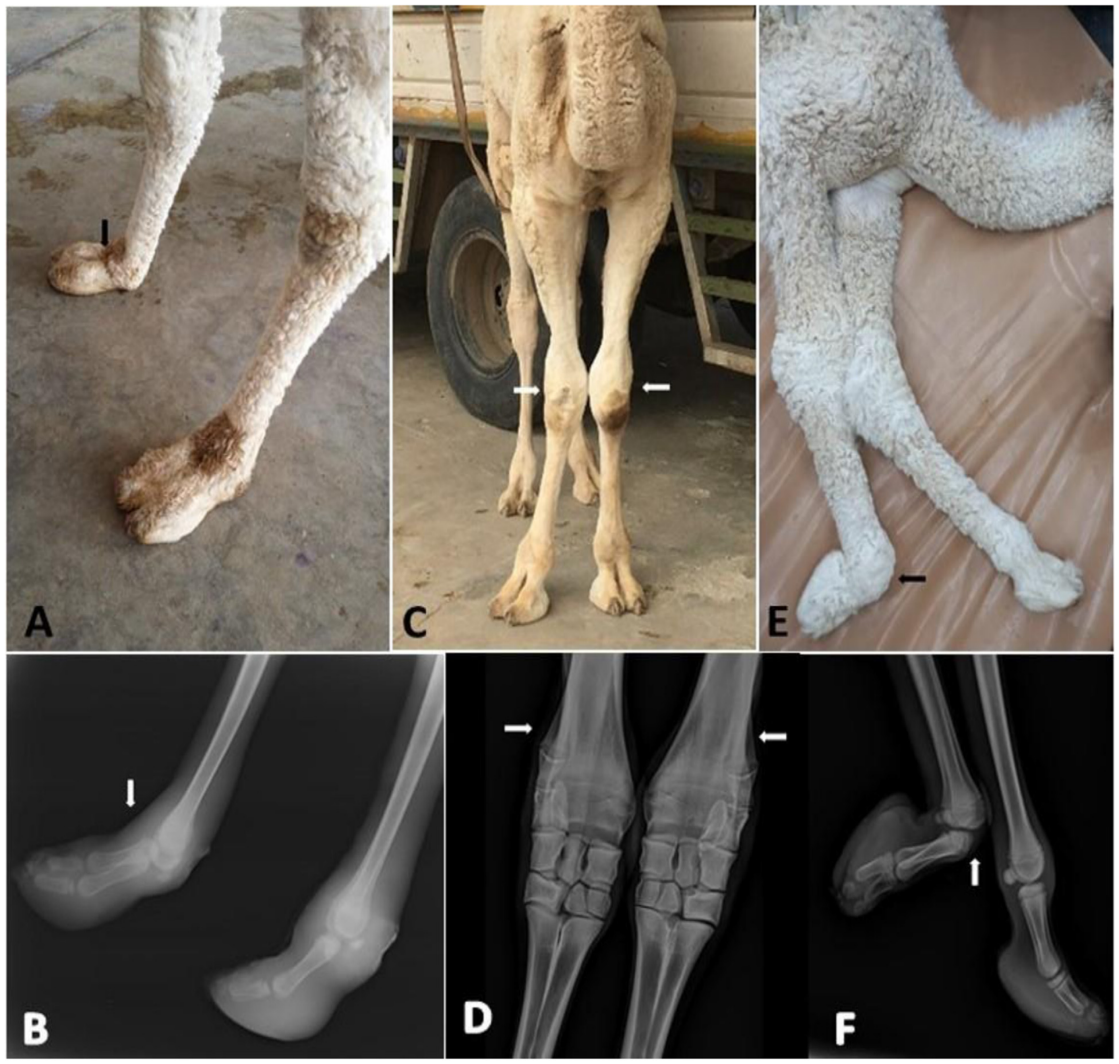
Clinical and radiographic presentations of flexural deformities. **(A)** Clinical appearance and **(B)** dorsopalmar radiograph of fetlock hyperextension in a 1-month-old calf, showing an increased joint angle (arrow). **(C)** Clinical view and **(D)** dorsopalmar radiograph of carpal valgus in an 8-month-old Ashaal calf; note the lateral deviation of the manus originating at the carpus (arrows). **(E)** Clinical appearance and **(F)** Lateromedial radiograph of fetlock hyperflexion (contracted tendons) in a 3-month-old female calf, showing a characteristically decreased joint angle (arrow).

**Figure 5:**
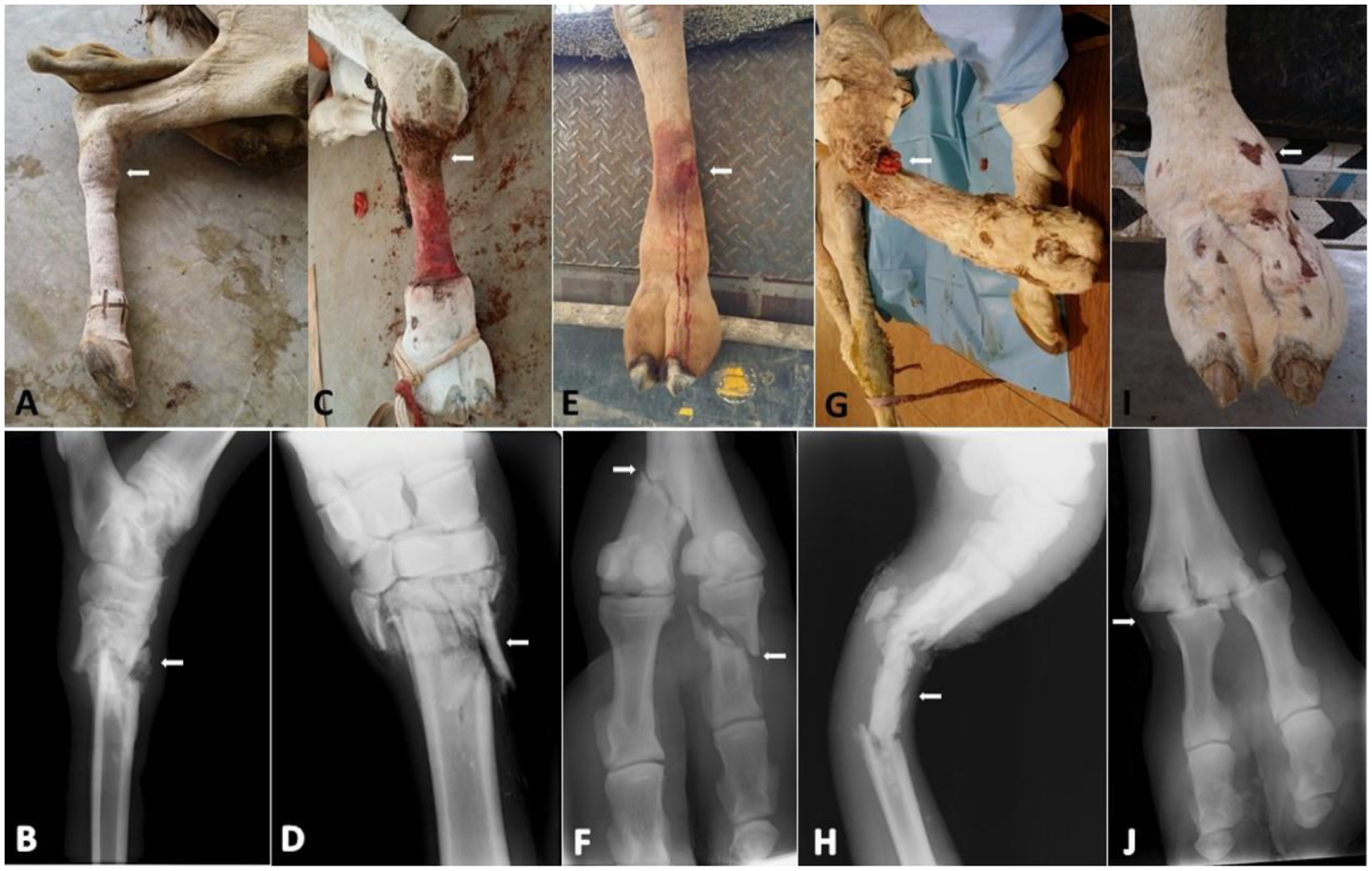
Diversity of fractures and dislocations in manus and pes, including the carpus and tarsus. **(A, B)** Complete metaphyseal metatarsal fracture in a 5-year-old Mejhem camel (arrow). **(C, D)** Dorsopalmar radiograph of a multiple epiphyseal metacarpal fracture (arrow) in a 6-year-old Ashaal camel. **(E, F)** Concurrent oblique fractures of the metacarpus and proximal phalanx (arrows) in a 7-year-old Wadeh camel. **(G, H)** Comminuted fracture (arrow) in a 2-month-old calf. **(I, J)** Complete luxation (dislocation) of the metacarpophalangeal joint (arrow) in a 5-year-old Ashaal camel.

**Figure 6:**
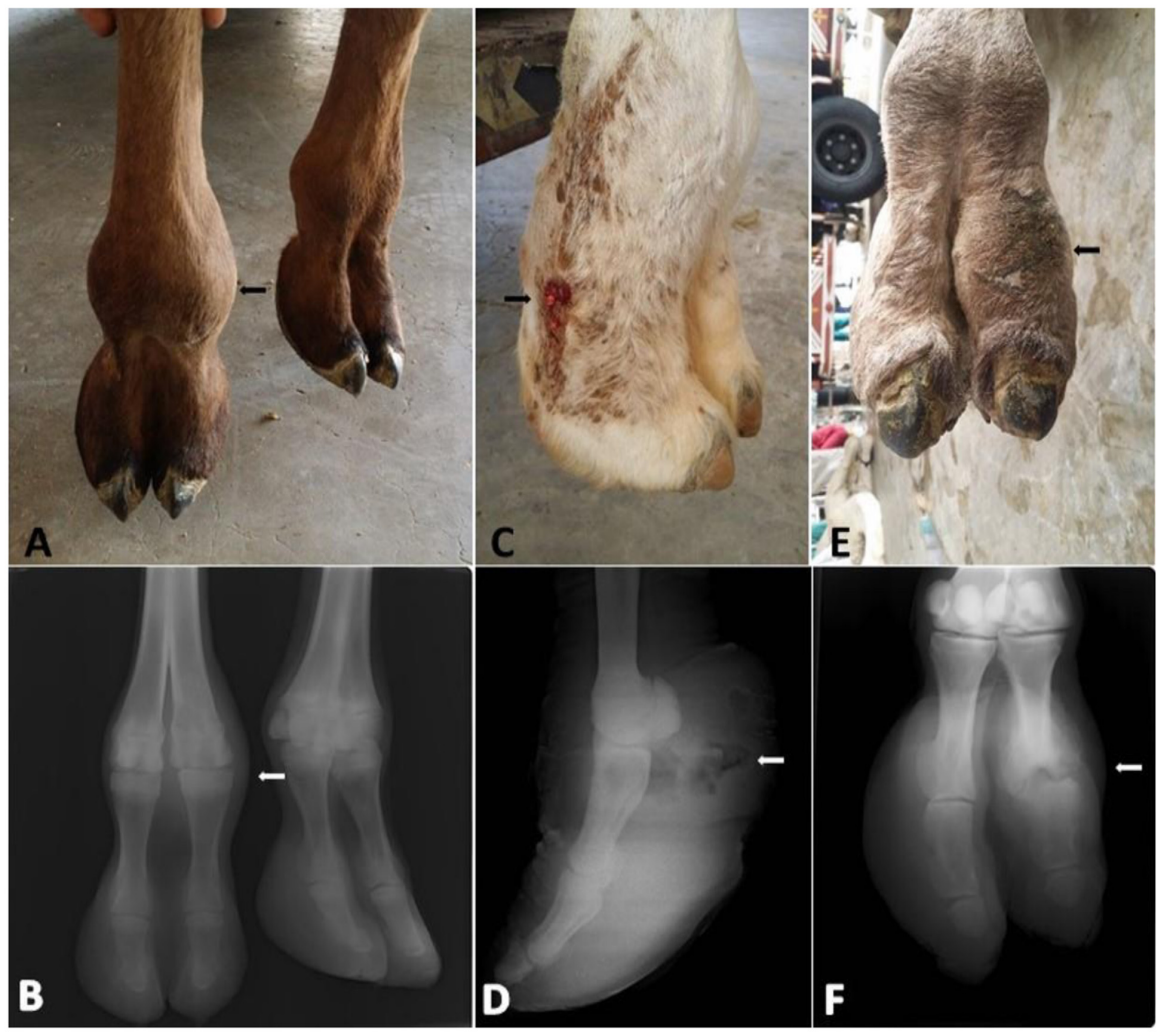
Radiographic characterization of acute, septic, and chronic arthritis. **(A, B)** Acute arthritis in an 8-month-old Asfar female camel; the radiograph reveals soft tissue outpouching of the joint capsule (arrow). **(C, D)** Septic arthritis in a 1-year-old Wadeh camel; note the radiolucent gas shadows within the joint space indicating anaerobic infection (arrow). **(E, F)** Chronic pastern joint arthritis in a 5-year-old Ashaal camel, characterized by significant osteophyte formation and cortical thickening (arrow).

**Figure 7:**
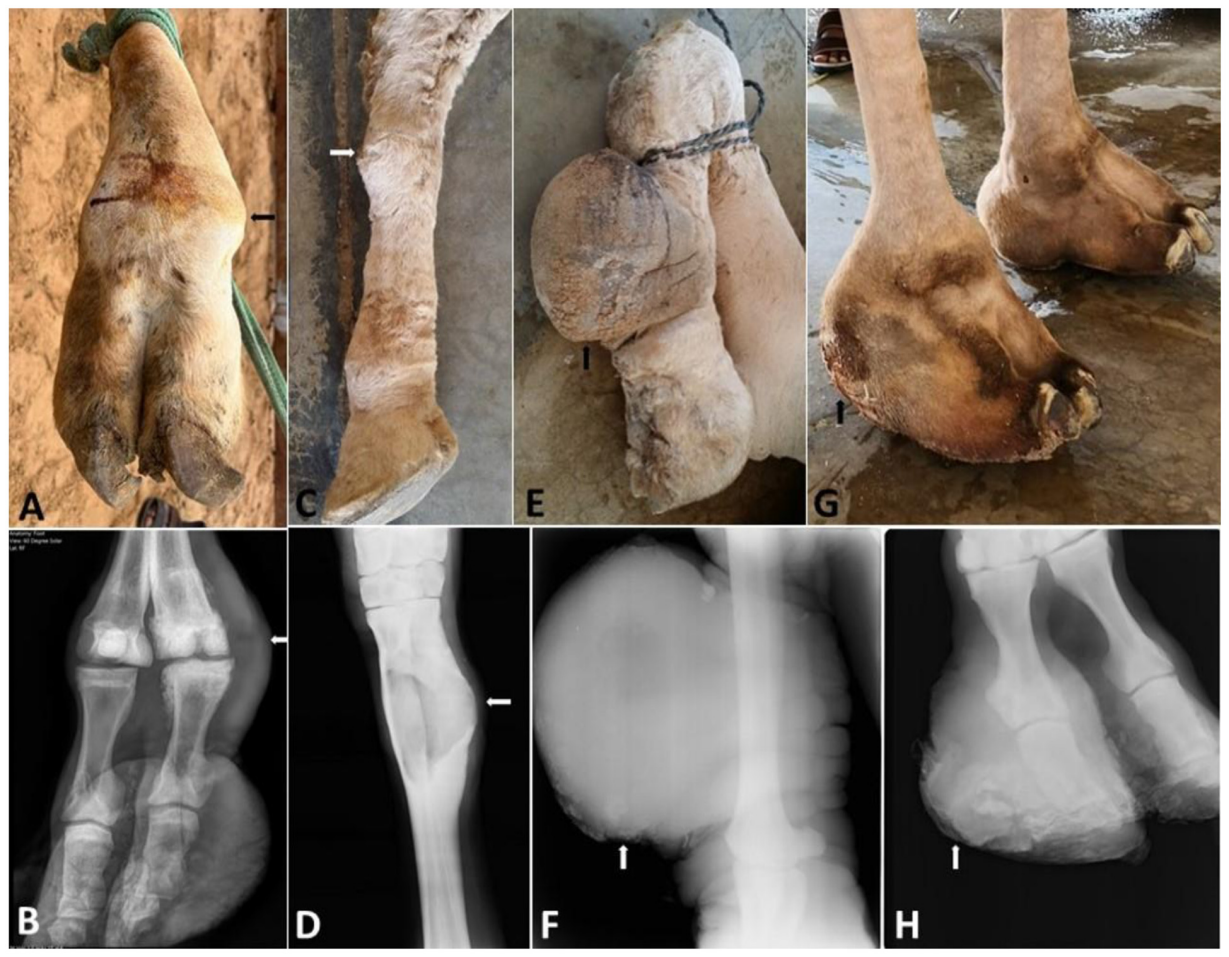
Proliferative bone lesions, metabolic changes, and soft tissue masses. **(A, B)** Bone exostosis of the first phalanx in a 7-year-old Wadeh camel, showing periarticular osteophytes and capsular thickening (arrow). **(C, D)** Metacarpal osteoporosis in a 2-year-old Asfar female, revealing a localized loss of bone density (arrow). **(E, F)** Soft tissue fibroma in a 4-year-old Wadeh camel; the radiograph shows a radiopaque cutaneous mass distinct from the underlying bone (arrow). **(G, H)** Bilateral fibrous bursitis of the fetlock secondary to congenital deformity, showing marked thickening of the footpad (arrows).

**Figure 8:**
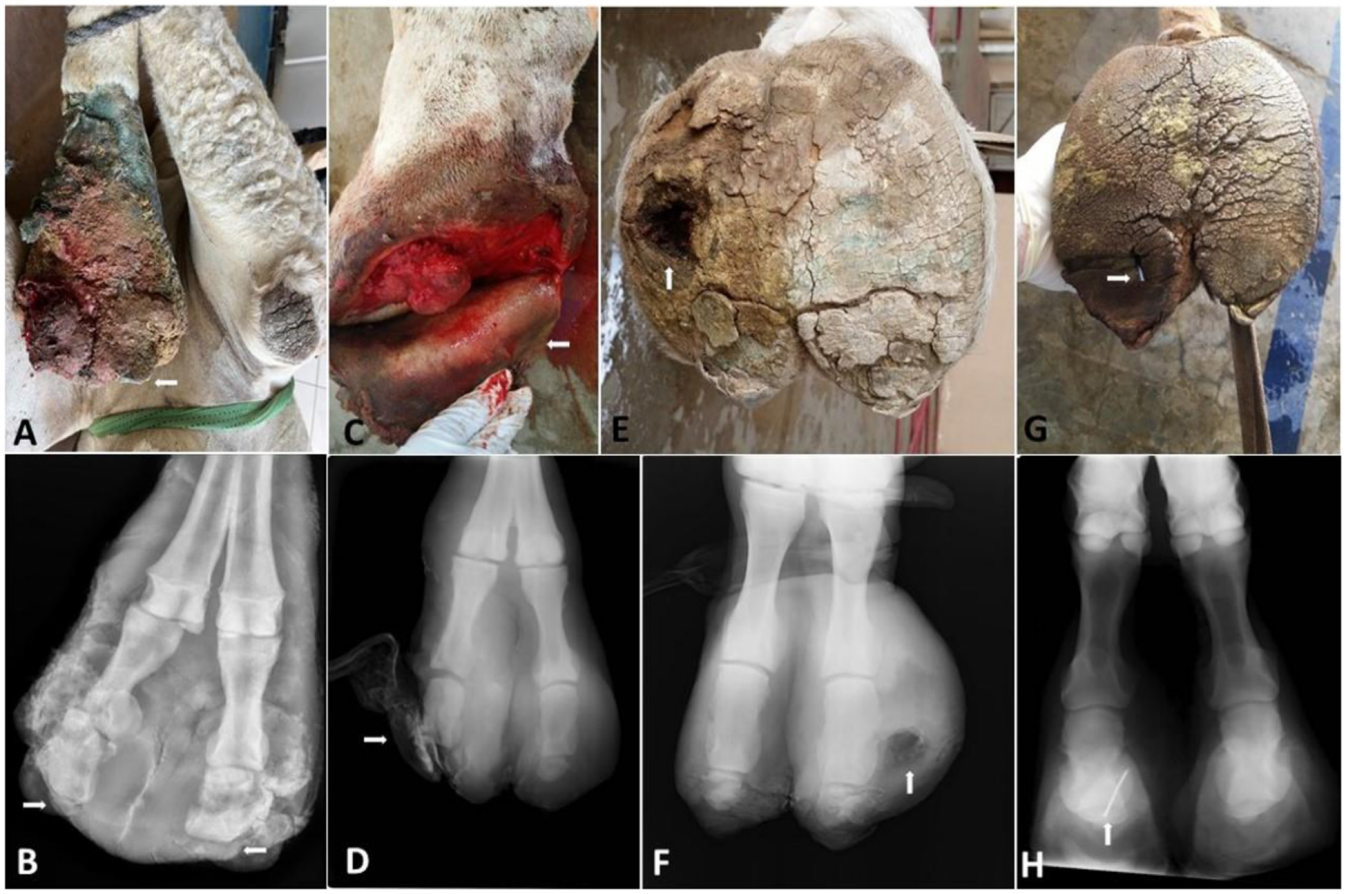
Trauma and infectious lesions of the footpad and phalanges. **(A, B)** Claw avulsion in a 6-year-old Wadeh camel, showing loss of the third phalanx (P3) on the right (arrow) and osteoporosis of P3 on the left (white arrow). **(C, D)** Sole avulsion with associated loss of P3 (arrow). **(E, F)** Deep footpad ulcer in a 4-year-old Wadeh camel; gas accumulation (arrow) indicates deep tissue infection. **(G, H)** Penetrating foreign body (nail) in a 5-year-old Asfar camel, showing the radiopaque object positioned cranial to the os pedal (arrow).

**Figure 9:**
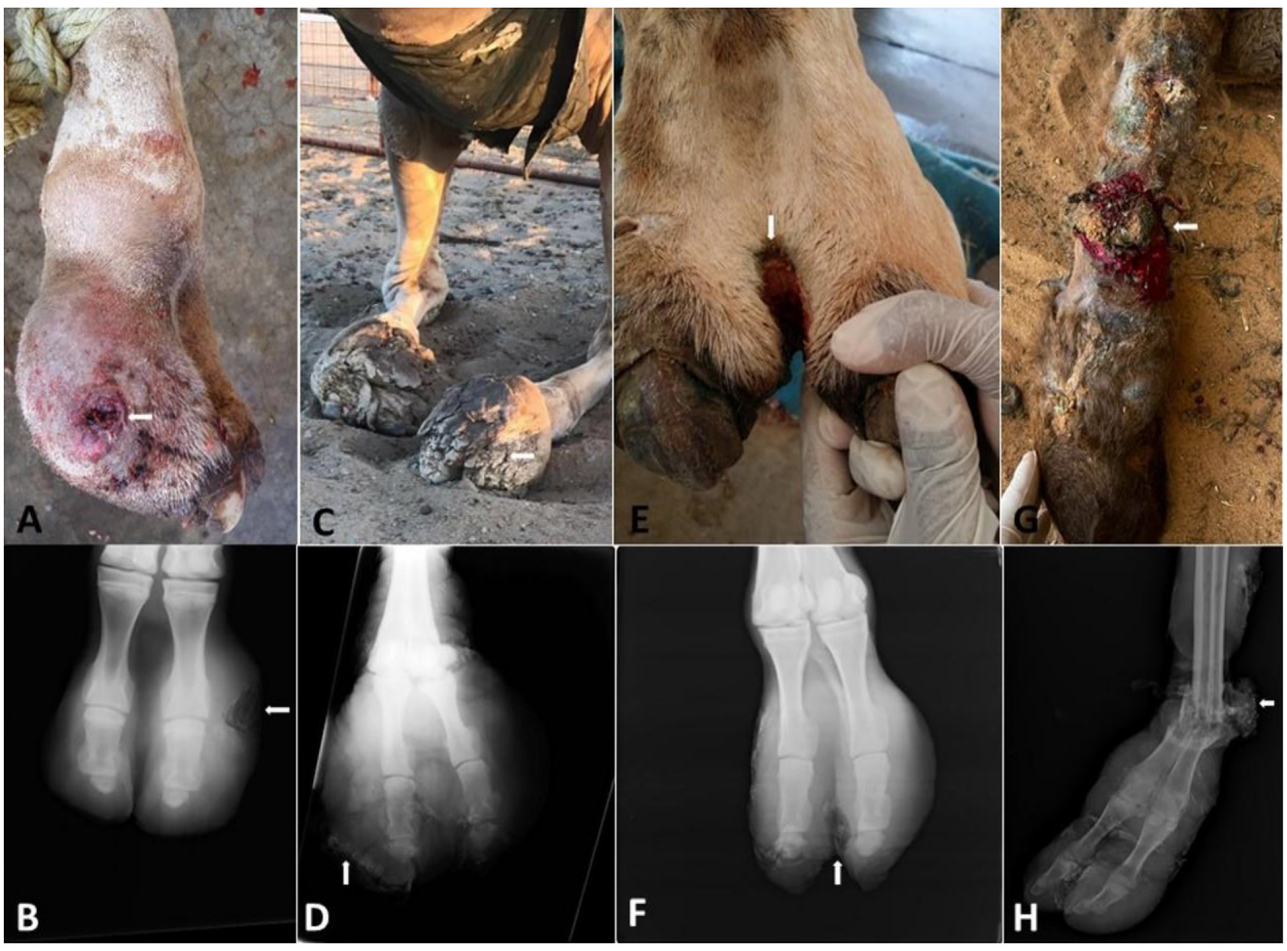
Chronic infections and integumentary disorders. **(A, B)** Infected wound in the left forelimb of a 7-year-old Wadeh camel with subcutaneous emphysema (arrow). **(C, D)** Hyperkeratosis of the fore claws in a 10-year-old camel, revealing a corrugated, radiopaque solar surface (arrows). **(E, F)** Interdigital dermatitis with associated ulceration and gas shadows (arrow). **(G, H)** Digit gangrene secondary to an open metatarsal fracture in a 4-month-old calf; note the exuberant, irregular callus and signs of osteomyelitis (arrow).

A total of 222 dromedary camels presenting with manus and pes lesions, including the carpus and tarsus were included in this retrospective study. Clinical and radiographic examinations identified 325 distinct lesions. Hard-tissue (osseous) lesions were predominant, comprising 203 (62.5%) of all diagnoses, while soft-tissue lesions accounted for the remaining 122 (37.5%). Detailed associations between lesion types, their clinical manifestations, and radiographic features are summarized in Table 3 and Figures 1–9.

**Table 3:** Frequency and distribution of clinical and radiographic findings of 325 manus and pes lesions, including the carpus and tarsus in a 222-camel cohort (Saudi Arabia, 2017–2025).

Category	Lesion type	Frequency (%)	Clinical and radiographic findings
Osseous tissue (62.5%)^b^	Fracture^a^	99 (30.46)	Clinical: crepitus, severe swelling, non-weight bearing Radiographic: cortical discontinuity, displacement
	Joint luxation^a^	59 (18.15)	Clinical: abnormal joint angulation, severe lameness Radiographic: loss of articular congruity
	Bony exostosis	19 (5.85)	Clinical: firm, non-painful chronic swelling Radiographic: periosteal reaction, new bone formation
	Polydactyly	7 (2.15)	Clinical: congenital extra digits Radiographic: supernumerary phalanges/meta-carpals/tarsals
	Bone deformity	5 (1.54)	Clinical: visible limb deviation (Varus/Valgus) Radiographic: abnormal bone curvature
	Osteoporosis	5 (1.54)	Clinical: stiff gait and lameness predisposition to pathological fractures Radiographic: decreased bone density, thin cortex
	Arthritis/Ankylosis	6 (1.85)	Clinical: joint effusion or loss of mobility Radiographic: joint space narrowing or bony bridging
	Brachydactyly	3 (0.92)	Clinical: congenital short digits Radiographic: abnormally shortened phalanges
Soft tissue (37.5%)	Fibroma**/**Tumors	28 (8.62)	Clinical: firm, circumscribed or invasive masses Radiographic: soft tissue opacity/density changes
	Flexural deformity	16 (4.92)	Clinical: knuckling at fetlock/pastern Radiographic: abnormal joint flexion angles
	Chronic septic wound	16 (4.92)	Clinical: drainage, granulation tissue Radiographic: localized soft tissue swelling
	Penetrating foreign body	11 (3.38)	Clinical: acute pain, puncture track in footpad (e.g., picked-up nail) Radiographic: visualized track or radiopaque object
	Footpad hyper keratin	10 (3.08)	Clinical: thickened, cracked skin Radiographic: minimal changes, superficial thickening
	Tendon lacerations	4 (1.23)	Clinical: dropped fetlock (flexor) or knuckling (extensor) Radiographic: focal soft tissue swelling
	Claw/Pad injuries	15 (4.62)	Clinical: avulsions, abscesses, or ulcers Radiographic: loss of normal nail/horn density
	Other soft tissue	22 (6.77)	Clinical: dermatitis, myiasis, bursitis, varicose, gangrene Radiographic: gas pockets (emphysema) or focal opacities

Values represent prevalence (%). Values within rows with different superscripts (a and b) differ significantly (p < 0.05).

Clinical and radiographic examinations of affected camels revealed various types of affections, including bony and soft tissues disorders affecting regions of the carpus, hock, and lower parts of the fore and hindlimbs, down to the footpads. Bony tissue affections included flexural deformity, bone deformity, brachydactyly, polydactyly, fractures, joint dislocations, arthritis and ankyloses osteoporosis, and bony exostosis. Soft tissue affections included here were old granulating wounds, clinical digital dermatitis, clinical bursitis, old infected wounds, tumors-like masses, picked-up nails, hyperkeratinization, footpad ulcers, footpad avulsions, overgrown claw nails, recent wounds, fibroma-like mass, myiasis, varicose, flexor and extensor tendon lacerations, pastern and foot loss, claw nail abscesses, claw nail avulsion, and footpad abscesses (Figures 3–9).

Our results identified a diverse range of 325 lesions, categorized by tissue type and clinical presentation (Table 3). Hard-tissue lesions were significantly more frequent than soft-tissue lesions (62.5%, OR = 3.40, 95% CI: 2.315–5.007, *p* = 0.001). Fractures were the most prevalent hard-tissue affection, diagnosed in 99 cases (30.5% of all lesions), demonstrating a significantly higher occurrence than all other affections (*p* = 0.001; OR: 0.19, 95% CI: 0.13–0.26; Figure 1). This was followed by joint dislocations (59 cases, 18.2%; *p* = 0.001; OR: 0.04, 95% CI: 0.03–0.07) and bony exostoses (19 cases, 5.8%; Figures 3, 4). Hard-tissue lesions were primarily characterized by structural or articular disruptions; for instance, fractures and joint dislocations were radiographically defined by cortical discontinuity and loss of joint congruity, correlating clinically with non-weight-bearing lameness (Grade 3) and crepitus. In contrast, bony exostoses and ankyloses presented as chronic, firm swellings with restricted mobility.

Soft-tissue lesions (37.5%) predominantly manifested as localized swellings, masses, or integumentary defects. Among soft-tissue affections, fibroma-like mass (18 cases, 5.5%), flexural deformities (16 cases, 4.9%), old septic wounds (16 cases, 4.9%), and picked-up nails (11 cases, 3.4%) were the most frequently encountered (Figure 5). Fibromas and tumors-like masses appeared as distinct soft-tissue opacities on radiographs, while conditions like hyperkeratinization and clinical digital dermatitis were purely clinical diagnoses localized to the footpad and interdigital skin. Functional deficits, such as flexural deformities and tendon lacerations, showed specific postural changes (e.g., knuckling) despite often having unremarkable radiographic bone findings, highlighting the necessity of combining both examination modalities for an accurate diagnosis. No significant variations in prevalence were found among these specific soft-tissue conditions (Table 3).

### Lameness severity and anatomical distribution

The clinical evaluation of the lameness lesions in the examined camel cohort revealed varying degrees of lameness based on the 0–3 grading scale (Table 1). This scoring system represents a non-validated clinical adaptation and is based strictly on functional movement, regardless of the underlying pathology. A total of 20 camels (6.15%) were identified as functionally sound (Grade 0), showing no detectable gait deviation. Among the animals exhibiting lameness, the most frequent presentation was severe lameness (Grade 3), which accounted for (54.77%) of the studied camels, representing cases where animals were reluctant to bear weight or move. Mild lameness (Grade 1) was observed in 62 camels (19.08%), characterized by subtle lameness difficult to observe at a walk but consistently apparent at a trot and slight shortening of stride. Moderate lameness (Grade 2) was observed in 65 camels (20%), characterized by consistent gait alterations and compensatory movements of the affected limb, such as head bobbing or pelvic hitching. Overall, these findings indicate a high prevalence of gait disorders within the examined camels, with the majority of affected animals falling into the (Grade 3) category.

Regarding distribution of affections between limbs, the forelimbs were significantly more prone to manus and pes lesions, including the carpus and tarsus compared to the hindlimbs (*p* = 0.001). Of the 325 manus and pes lesions, including the carpus and tarsus, 192 (59.08%) exhibited forelimb affections, while 133 (40.92%) involved the hindlimbs. Logistic regression identified that camels were 1.4 times more likely to present with lesions in the forelimbs than in the hindlimbs (OR: 1.44; 95% CI: 1.16–1.80). A systematic comparison revealed that while both limbs followed a similar distal-to-proximal distribution of lesions, the forelimb exhibited a significantly higher burden of osseous affections, particularly within the cannon region (*n* = 69), compared to the hindlimb (*n* = 38). Interestingly, soft tissue involvement remained relatively balanced between the forelimb (*n* = 67) and hindlimb (*n* = 55), suggesting that the increased incidence of lameness in the forelimb (59.08%) is primarily driven by skeletal rather than soft-tissue pathologies (Table 4).

**Table 4:** Comparative distribution of 325 manus lesions, including the carpus vs. 222 pes lesions, including the tarsus-camel cohort examined in Saudi Arabia from 2017 to 2025.

Region	Forelimb	Hindlimb	*p*-Value	Odds ratio	95% CI
Carpus/Tarsus	6 (3.05%)	3 (2.26%)	0.745	1.36	0.33–5.54
Cannon region	69 (35.03%)	38 (28.57%)	0.268	1.35	0.84–2.17
Fetlock region	46 (23.35%)	31 (23.31%)	1.000	1.00	0.60–1.69
Pastern region	39 (19.80%)	29 (21.80%)	0.761	0.89	0.52–1.52
Coffin region	28 (14.21%)	24 (18.05%)	0.434	0.75	0.41–1.37
Claw/Footpad	9 (4.57%)	8 (6.02%)	0.742	0.75	0.28–1.99
Total lesions	192 (59.08%)	133 (40.92%)	0.001	1.44	1.16–1.80

### Univariate and multivariate analysis of risk factors

Several animal-related factors were significantly associated with the prevalence of manus and pes lesions, including the carpus and tarsus in camels (Table 5). Juveniles had higher odds of affection compared to calves and adults (*p* = 0.017; OR: 1.6; 95% CI: 1.094–2.315). Female camels were more frequently affected than males (*p* = 0.01; OR: 1.66; 95% CI: 1.134–2.404). Camels with a body weight between 150 to 500 kg were at the highest risk (*p* = 0.001; OR: 4.0; 95% CI: 2.694–5.944). Furthermore, the Wadeh breed was significantly more predisposed than other breeds (*p* = 0.007; OR: 2.055; 95% CI: 1.368–3.078). An association was documented between the manus and pes lesions, including the carpus and tarsus and their acquisition (*p* = 0.0001; OR: 91.6; 95% CI: 47.51–174.3). The vast majority of lesions were acquired rather than congenital (90.5 vs. 9.5%; *p* = 0.001; OR: 91.6).

**Table 5:** Classification of individual camels as positive or negative for surgical affections with respect to different risk factors.

Variable	Category	Positive (*n* = 222)	*p*-Value	Odds ratio (OR)	95% confidence interval (CI)
Age	Calve	61 (27.48%)			
	Juvenile	98 (44.14%)	0.017	1.60	1.094–2.315
	Adult	63 (28.38%)			
Gender	Male	97 (43.7%)			
	Female	125 (56.3%)	0.01	1.66	1.134–2.404
Body weight	< 200 kg	63 (28.38%)			
	200–500 kg	148 (66.67%)	0.001	4.0	2.694–5.944
	>500 kg	11 (4.95)			
Breed	Wadeh	87 (39.19%)	0.007	2.055	1.368–3.078
	Mejhem	53 (23.88%)			
	Ashal	42 (18.92%)			
	Asfar	40 (18.01%)			
Occurrence	Congenital	21			
	Acquired	201	0.0001	91.6	47.51 −174.3

The forelimbs were significantly more prone to manus and pes lesions, including the carpus and tarsus than the hindlimbs (*p* = 0.0001; OR: 3.69). Clinical swelling was present in 84.7% of cases and was strongly associated with manus and pes lesions, including the carpus and tarsus disorders (*p* = 0.001; OR: 30.57; 95% CI: 18.24–51.64). There was a significant association between manus and pes lesions, including the carpus and tarsus and tissue type (hard vs. soft), with an odds ratio of 3.40 (95% CI: 2.315–5.007; *p* = 0.001). Specifically, hard-tissue affections were more frequent, occurring in 144 camels, as compared to 78 with soft-tissue affections. Most camels presented with a single lesion (67.11%), though significant variation was observed in the number of lesions per animal (*p* = 0.05; OR: 9.0; 95% CI: 4.3–9.94). Among camels with multiple affections, 53 had two, 14 had three, five had four, and one had five. The multivariate statistical analysis is summarized in Table 6, including body weight, sex, and age.

**Table 6:** Final logistic regression model for risk factors associated with surgical affections of the limb in camels in Saudi Arabia.

Variable	β	S.E.	DF	*p*-Value	OR	95% CI
Weight	1.801	0.850	1	0.034	0.165	0.031–0.83
Gender (female)	1.805	0.668	1	0.007	6.082	1.641–22.53
Age (juvenile)	0.693	0.289	1	0.016	0.500	0.284–0.88
Constant	0.153	0.223	1	0.493	1.165	

**β**, regression coefficient; SE: standard error; DF, degree of freedom; p-value, significance level; OR, odds ratio; 95% CI, confidence interval at 95%.

### Prognosis and treatment outcomes

The overall prognosis was favorable, with a significant majority of camels (146 vs. 76) responding positively to treatment (*p* = 0.001; OR: 3.69; 95% CI: 2.497–5.451). Treatment failure was significantly associated with the complexity of the case and the number of manus and pes lesions, including the carpus and tarsus per camel (*p* = 0.034). This association followed a clear trend; the likelihood of treatment failure was highest in camels with multiple affections and decreased as the number of affections decreased (*p* = 0.05; OR: 9.0; 95% CI: 4.3–9.94; Table 7).

**Table 7:** Association between surgical affections and clinical variables in a 222-camel cohort.

Variable	Category	Affected camels (*n* = 222)	*p*-Value	Odds ratio	95% CI
Response to treatment	Responded	146 (65.77%)	0.0001	3.690	2.497–5.451
	Hopeless case	76 (34.23%)			
Swelling	Swollen	188 (84.69%)	0.0001	30.57	18.24–51.64
	Not swollen	34 (15.31%)			
Target tissue (325 affections)	Soft structure	122/325 (37.5%)			
	Hard structure	203/325 (62.5%)	0.0001	2.769	2.005–3.814
Number of affections in each animal	One affection	149 (67.11%)	0.05	9.0	4.3–9.94
	Two affections	53 (23.87%)			
	Three affections	14 (6.32%)			
	Four affections	5 (2.25%)			
	Five affections	1 (0.45%)			

## Discussion

Lameness is a paramount welfare and economic concern in the camel industry, ranked as the third most significant health issue affecting dromedaries (1, 19). The high prevalence of manus and pes lesions, including the carpus and tarsus observed in this large-scale retrospective study underscores their substantial impact on animal wellbeing, productivity, and clinical management. Subclinical conditions, if prolonged, may progress to chronic disease, reinforcing the necessity of early diagnosis and timely intervention to mitigate long-term economic losses (7, 20).

Dromedary camels possess unique biomechanical and anatomical adaptations that distinguish their lameness presentation from that of other large ungulates. Unlike cattle, which primarily employ diagonal gait, camels are natural pacers, characterized by the synchronized movement of the ipsilateral fore and hind limbs (1). This lateral gait, combined with a secondary digitigrade stance and the presence of broad, fibroelastic foot pads, is an evolutionary adaptation for efficient locomotion on unstable desert substrates. These features significantly impact weight distribution and the minimization of mechanical impact during movement (3, 19). Consequently, clinical markers of lameness—such as the timing of peak vertical ground reaction forces or the expression of compensatory trunk movements—may be more subtle or manifest differently in the camelid pace compared to the bovine walk. By recognizing these species-specific dynamics, the adapted scoring system used in this study aimed to capture functional irregularities within the framework of the camel's unique locomotor profile. This scoring system is a modified, non-validated adaptation of bovine scales, necessitated by the absence of a standardized camel-specific tool.

The disproportionately high prevalence of severe lameness (Grade 3; 54.77%) observed in this cohort reflects the nature of the study population, which primarily consisted of camels presented for emergency surgical intervention or specialized orthopedic care. Unlike field-based surveys, where mild (Grade 1) or subclinical gait alterations typically predominate, our findings are skewed toward acute traumatic injuries, such as fractures, joint dislocations, and septic arthritis, that directly compromise skeletal integrity, joint stability, soft tissue support, or weight-bearing structures of the limb and necessitate immediate clinical attention (18, 20). The clinical severity of septic arthritis and foreign body penetrations (e.g., “picked up” nails) in camels is particularly noteworthy; the unique anatomy of the camel's digital cushion allows for deep tracking of infection, often leading to rapid structural compromise and intense pain. Conversely, chronic conditions like osteoporosis, flexural deformities, and bony exostosis resulted in more moderate, progressive gait alterations (Grade 2). These conditions represent mechanical or degenerative changes rather than acute structural failure (1, 3). Interestingly, congenital anomalies and mild footpad hyperkeratinization were often subclinical (Grade 0), suggesting that while these lesions are morphologically evident, they do not always translate into functional lameness unless complicated by secondary trauma or infection (11).

Osseous lesions represented the majority of manus and pes lesions, including the carpus and tarsus (62.5%), reflecting the biomechanical vulnerability of the digits, which serve as the primary weight-bearing and shock-absorbing structures during locomotion in desert environments (21–23). Continuous exposure to uneven terrain and high-impact forces likely contributes to the high frequency of fractures, dislocations, and exostoses. These findings further emphasize the diagnostic value of radiography, which is particularly effective for identifying skeletal lesions that may be underestimated during clinical examination alone. Similar observations have been reported in previous studies (18, 20, 31).

The high prevalence of fractures (30.5%) and dislocations (18.2%) underscores the substantial traumatic and stress-related forces acting on the camel's manus and pes, including the carpus and tarsus. The considerable body mass of camels, combined with locomotion on challenging terrain and intensive activities such as racing and rearing, predisposes the fetlock and other distal joints to both acute and chronic injuries (17, 23). Forelimb lesions were significantly more common than hindlimb lesions, which are anatomically and physiologically expected, as camels bear a greater proportion of their body weight on the forelimbs. This uneven weight distribution is a key etiological factor in camel lameness (24–26).

Our results demonstrated a significant predilection for lesions in the forelimbs compared to the hindlimbs (59.08 vs. 40.92%; *p* = 0.001 OR: 1.44), which aligns with the biomechanical loading patterns characteristic of dromedary camels. During locomotion and static stance, camels typically bear approximately 60%−65% of their total body weight on their forelimbs (1, 3). This increased vertical loading and concussion during locomotion likely predisposes the carpal joint and distal forelimb structures to greater concussive stress and cumulative trauma than the tarsus or hindlimb regions. The higher incidence of forelimb injuries may also be attributed to the animal's pacing gait, where the splayed-toed anatomy of the manus absorbs significant impact on varied terrains, potentially leading to the frequent foot wounds and fetlock pathologies observed in our sample.

Analysis of risk factors provides critical insights for preventive management. Juvenile camels were more frequently affected than calves or adults (*p* = 0.017; OR: 1.6; 95% CI: 1.094–2.315), likely due to skeletal immaturity, increased activity, and developing coordination, which increase susceptibility to traumatic injury (19, 27). Regarding biological sex, female camels showed higher odds of developing manus and pes lesions, including the carpus and tarsus compared to males (*p* = 0.01; OR = 1.66; 95% CI: 1.134–2.404). While this association is statistically significant, potential explanations may include management factors, such as higher stocking densities or longer retention of females within herds, whereas males are often slaughtered at a younger age for meat production. In addition, biomechanical or reproductive-related factors may contribute to this observed predisposition (2, 13, 24). However, these possibilities remain speculative and should be considered as hypotheses requiring further investigation.

The significant association between body weight and manus and pes lesions, including the carpus and tarsus is particularly noteworthy. This relationship likely reflects a critical “risk window” where increasing body mass generates substantial forces at a time when skeletal maturity and structural resilience are not yet fully developed (28, 29). Additionally, Wadeh camels showed a significant breed predisposition (*p* = 0.007; OR = 2.055; 95% CI: 1.368–3.078). This finding may be partially explained by the predominance of this breed in Saudi Arabia due to its high productive value, aligning with trends documented in other regional studies (30, 31).

The vast majority of manus and pes lesions, including the carpus and tarsus were classified as acquired rather than congenital (*p* = 0.0001), indicating that postnatal external factors play a dominant role in their pathogenesis. This suggests traumatic events, management practices, and environmental influences, rather than developmental defects, primarily drive manus and pes, including the carpus and tarsus pathology in camels. Consequently, improving husbandry standards, implementing routine limb and hoof examinations, and mitigating environmental hazards are key strategies for reducing the incidence of these lesions.

The findings of this study further confirm the essential role of radiography in the clinical management of camel lameness. Many osseous lesions may remain undetected during early stages and progress to chronic, severe pathological changes. While clinical examination identifies soft tissues swelling and gross deformities, radiography is critical for the definitive diagnosis of specific osseous skeletal lesions—including fractures, exostoses, and dislocations—and for accurately determining lesion severity to guide timely surgical or medical intervention. By transforming clinical suspicion into a precise diagnosis, radiography enables more accurate prognostication. Accordingly, the combined clinical and radiographic approach applied in this study allows most manus and pes lesions, including the carpus and tarsus to be reliably diagnosed in practice (4, 6–8).

Overall, the prognosis for manus and pes lesions, including the carpus and tarsus in camels was favorable, with most cases responding positively to treatment (*p* = 0.001). However, a strong inverse relationship was observed between the number of concurrent lesions and treatment success. While isolated injuries typically carry a good prognosis, camels presenting with multiple simultaneous pathologies (e.g., fractures combined with septic wounds) often exhibit compromised healing due to severe trauma or systemic involvement. Clinicians should exercise increased caution when managing such cases, as they require intensified monitoring and tailored therapeutic strategies. This provides veterinarians with an evidence-based prognostic indicator at the time of initial examination (11, 12, 17).

This study's limitations include its retrospective, single-center design, which likely introduced referral bias toward more severe cases, potentially overestimating prevalence compared to the general population. The analysis did not account for external variables such as husbandry systems, nutrition, or workload, nor did it evaluate the genetic or conformational drivers of breed-related susceptibility. Methodologically, the study relied on a subjective lameness scoring system adapted from bovine scales—which may not fully capture the dromedary's unique biomechanics—without formal kinematic validation or inter-observer reliability testing. Finally, the lack of histopathological or cytological confirmation for soft tissue lesions and certain clinical diagnoses suggests that future prospective, multicenter studies are needed to establish more definitive diagnostic standards and improve external validity.

In conclusion, injuries of the manus and pes, including the carpus and tarsus in camels represent a significant welfare concern with notable economic implications. Within the clinical decision-making framework, radiography played an essential role in supporting definitive diagnosis, guiding treatment planning, and informing prognosis. The identification of relevant risk factors, particularly for juvenile camels and the Wadeh breed, will facilitate the development of targeted preventive strategies and trauma-minimization measures.

## Data Availability

The original contributions presented in the study are included in the article/supplementary material, further inquiries can be directed to the corresponding author.
